# Near-Term Anti-CD25 Monoclonal Antibody Administration Protects Murine Liver from Ischemia-Reperfusion Injury Due to Reduced Numbers of CD4^+^ T Cells

**DOI:** 10.1371/journal.pone.0106892

**Published:** 2014-09-04

**Authors:** Jinghui Yang, Xiaoyu Wang, Shaohua Song, Fang Liu, Zhiren Fu, Quanxing Wang

**Affiliations:** 1 Department of Organ Transplantation, Shanghai ChangZheng Hospital, Second Military Medical University, Shanghai, China; 2 National Key Laboratory of Medical Immunology, Second Military Medical University, Shanghai, China; University of Navarra School of Medicine and Center for Applied Medical Research (CIMA), Spain

## Abstract

**Background:**

CD4^+^ T cell is acknowledged as a key factor in the initiation phase of liver ischemia reperfusion injury. The purpose of current study is to demonstrate the effect of antecedent near-term anti-CD25 monoclonal antibody treatment on IR-induced liver injury by modulation of CD4^+^ T cells.

**Methods:**

70% liver warm IR was induced in male C57BL/6 mice after anti-CD25 mAb or non-specific IgG administration. Liver function, histological damage, *in* *vitro* Proliferation, FACS, cytokine production, and immunofluorescence were assessed to evaluate the impact of antecedent near-term PC61 treatment on IR-induced liver injury.

**Results:**

After 70% liver ischemia, mice preconditioned with PC61 displayed significantly preserved liver function as characterized by less histological damage and reduced serum enzymes level. Mechanistic studies revealed that the protection effect of anti-CD25 mAb was associated with ameliorated intrahepatic inflammatory milieu and reduced CD4^+^ T lymphocytes as manifested by the decrease of proinflammatory cytokine production (less expression of TNF-α, IFN-γ, IL-2, and IL-6) and the lower CD4/CD8 proportion.

**Conclusions:**

Our results provide first line of evidence indicating that near-term treatment with anti-CD25 monoclonal antibody might provide protection for livers against IR-induced injury by reducing CD4^+^ T cells, but not influencing functional Treg population. Therefore, our results demonstrate a potential function of anti-CD25 monoclonal antibody which was neglected in the past, and may be helpful in various clinical conditions, particularly in liver and kidney transplantations.

## Introduction

Liver ischemia reperfusion injury (IRI) is a clinically relevant condition that occurs during resection surgery, trauma, hypovolemic shock, or transplantation when liver is transiently deprived of oxygen and reoxygenated. These conditions result in hepatic dysfunction and failure as well as remote organ injury [1]. The pathophysiology of liver IRI includes direct cellular damage as the result of the ischemic insult as well as delayed dysfunction and damages that result from activation of inflammatory pathways. Clinical and experimental data have established that up to 10% early graft dysfunction and higher incidence of both acute and chronic rejection are associated with IRI, and therefore, it dampens the long-term graft survival [2]. Hepatic injuries caused by IRI are now recognized as a result of highly complex mechanisms, among which, the role of T lymphocytes has been proved of great importance and as a key mediator of IRI [3]. Nude, SCID, RAG1^–/–^, TCRαβ^–/–^, and CD4^–/–^ mice have all been shown to be protected from IRI. Experiments in which the protected phenotype of nude mice has been reversed by adoptive transfer of CD4^+^, but not CD8^+^ T cells, have been published in both renal and liver ischemia models [4]–[7]. These studies indicate that T lymphocyte is the key regulator in initiating and propagating the injury response.

One may therefore speculate whether a reduction in T lymphocytes may reduce the incidence and severity of IR-induced complications. IL-2R-specific monoclonal antibody (mAb) was used in clinics to inhibit most of the IL-2/IL-2R interaction for a considerable time, and prevented rejection in organ transplantation [8]. It acts as an antagonist at the interleukin-2 (IL-2) binding site of the p55 subunit (Tac, antigen) of the high affinity IL-2 receptor (CD25) on the surface of the activated T lymphocytes and inhibits the binding of serum IL-2 to CD25, there by inhibiting the proliferation of activated T cells and subsequent release of cytokines [9], [10]. However, one of the most important conflicts is that the current interventions targeting the IL-2R through anti-CD25 mAb can reduce the number and function of Treg cells, and eventually aggravate the IR injury [11], [12].

In the present study, we sought to elucidate whether near-term intervention targeting the IL-2R through anti-CD25 mAb might compromise the number or function of Treg cells in the liver. Our data showed the effect of anti-CD25 mAb and the role of Treg during acute liver inflammatory injury induced by IR and therefore indicated another mechanism of clinical good performance of anti-CD25 mAb in transplantations besides organ tolerance induction.

## Materials and Methods

### Animals and ethics statement

Male C57BL/6 mice (8–12 wk, weight 20–26 g) were obtained from Joint Ventures Sipper BK Experimental Animal Company (Shanghai, China). All animal experiments were performed in accordance with the National Institutes of Health Guide for the Care and Use of Laboratory Animals, with the approval of the Scientific Investigation Board of Second Military Medical University (Shanghai, China).

### Induction of liver IR

Mice were anesthetized with sodium pentobarbital (50 mg/kg, intraperitoneally, IP). After a midline laparotomy, an atraumatic clamp (Shanghai Medical Instruments, Shanghai, China) was used to interrupt blood supply to the left lateral and median lobes of the liver (70%). After 60 minutes of partial hepatic ischemia, the clamp was removed to initiate hepatic reperfusion. Mice with sham surgery (no interruption of the hepatic blood flow) were used as controls. Body temperature was maintained with an adjustable heating pad at 37°C. Some mice were injected with 300 µg/mouse of anti-mouse CD25 mAb (PC61) or control IgG 60 minutes before the ischemia insult. Mice were sacrificed after the indicated periods of reperfusion, and blood and samples of the livers were taken for analysis.

### Assessment of liver function

Serum AST and ALT levels were determined to assess the liver function by using a standard Modular Auto analyzer at the Central Laboratory, Changhai Hospital, Shanghai.

### Histopathological examination

Haematoxylin and eosin-stained slides were prepared from routinely processed excised specimens fixed in 10% buffered formalin and processed in paraffin. Three representative sections from each liver were scored. At least 10 high-power fields (×200) per section were examined for each sample. Histological examination was performed by two pathologists in a blinded fashion.

### FACS analysis

Fleshly obtained hepatic nonparenchymal cells, spleen cells and Peripheral blood cells were stained with the following antibodies: 7-AAD, CD45-PE, CD45-FITC, CD3-FITC, CD3-APC, CD4-PE, CD8α-PE, CD25-APC, FoxP3-APC, CD11b-PE, F4/80-APC, NK1.1-PE, B220-APC, CD19-PE, GR-1-FITC (BD Biosciences, San Diego, CA). Then the cells were washed three times, resuspended in 500 µl of PBS. Data acquired by Accuri C6 (BD Biosciences) were analyzed using CFlow software (BD Biosciences).

### In Vitro Proliferation Assays

Liver samples were homogenized in lysis buffer (10 mM of HEPES [pH 7.9], 150 mM of NaCl, 1 mM of EDTA, 0.6% NP40, 1 mM of PMSF, 1 µg/mL of leupeptin, 5 µg/mL of aprotonin, 10 µg/mL of SBTI, and 1 µg/mL of pepstatin). Samples were then sonicated and incubated for 30 minutes on ice. Cellular debris was removed by centrifugation at 10,000 rpm. Protein concentrations of each sample were determined.

Splenocytes were labeled with 5 mM 5-(and-6)-carboxyfluorescein diacetate, succinimidyl ester (CFSE; Invitrogen) and co-cultured with samples’ protein in 5% CO_2_ at 37°C. After 72 hours proliferation was assessed by flow Cytometry. Viable CFSE-labeled T cells were gated using 7-AAD, CD4-FITC and CD8α-PE (BD Biosciences) staining and the percentage of dividing cells was determined using the CFlow software.

### Tissue Immunofluorescent Staining

Sixty minutes after reperfusion, fresh liver were cut into 10 µm sections, mounted on glass slides, and stored at −20°C. At the time of staining, frozen sections were fixed in 4% paraformaldehyde and then treated with blocking solution containing 2 mg/ml normal donkey serum (Jackson ImmunoResearch, West Grove, PA) and 0.2% Triton X-100 in PBS for 30 min at 4°C. All incubations were carried out in a light protected, humidified chamber. After the excess blocking solution was drained, the tissues were stained overnight at 4°C with primary mAb against PE-conjugated CD4 (1∶1000 dilution; L3T4, eBioscience, San Diego, CA). After incubation with primary antibody, slides were washed twice with PBS and digitally imaged with a fluorescent microscope.

### Real-time RT-PCR analysis

Total liver RNA was extracted using TRIzol (Invitrogen) reagent according to the manufacturer’s instructions. cDNA was synthesized using oligo d(T) (Applied Biosystems) and a SuperScript III Reverse Transcriptase Kit (Invitrogen). A StepOne Real-Time PCR System (Applied Biosystems) and a SYBR RT-PCR kit (Takara) were used for quantitative real-time RT-PCR analysis. All reactions were conducted in a 20 µl reaction volume in triplicate. The relative expression levels for a target gene were normalized by GAPDH. Specificity was verified by melting curve analysis and agarose gel electrophoresis. Primers used RT-PCR analysis are: TNF-α (5′-AAG CCT GTA GCC CAC GTC GTA-3′; 5′-GGC ACC ACT AGT TGG TTG TCT TTG-3′); IL-2 (5′-CCA TGA TGC TCA CGT TTA AAT TTT-3′; 5′-CAT TTT CCA GGC ACT GGA GAT G-3′); IL-6 (5′-ACA ACC ACG GCC TTC CCT ACT T-3′; 5′-CAC GAT TTC CCA GAG AAC ATG TG-3′); IFN-γ (5′-GAA CTG GCA AAA GGA TGG TGA-3′; 5′-TGT GGG TTG TTG ACC TCA AAC-3′); and GAPDH (5′-TGA CCA CAG TCC ATG CCA TC-3′; 5′-GAC GGA CAC ATT GGG GGT AG-3′). Data were analyzed using the comparative C_t_ (2^−ΔΔCt^) method.

### Statistical analysis

The computer software GraphPad Prism 5 (GraphPad Software, La Jolla, CA) was used for data analysis. Data derived from two groups were analyzed using an unpaired Student’s *t* test or a Mann–Whitney test (two tailed). ANOVA was performed for comparisons of three groups. All data are expressed as mean ± SD. In all cases, p<0.05 was considered with statistical significance.

## Results

### CD4^+^ T cells in liver tissue expressed CD25 after 12 h of reperfusion

**Figure 1:**
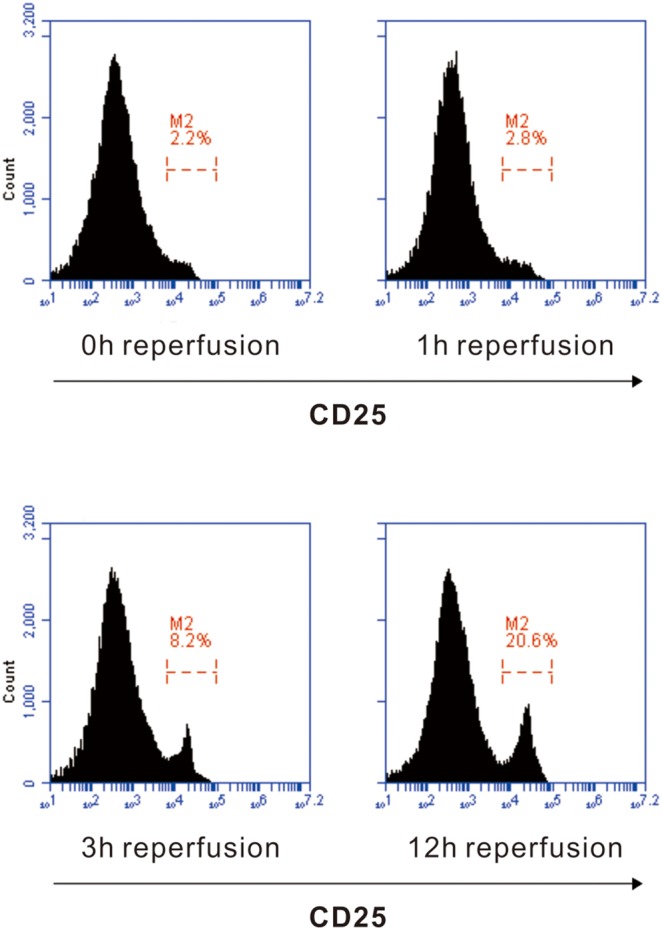
Detection of CD4^+^ T cell activation by up-regulation of CD25. After partial ischemia and 0, 1, 6, 12 hours reperfusion, CD4^+^ T cells were gated, and the proportion of CD4^+^CD25^+^ T cells were quantitated by FACS.

To confirm that reperfusion injury may activate T cells despite the absence of antigenic stimulation, we determined the level of CD25^+^ T cells in liver tissue after 0, 1, 6, 12 hours of reperfusion. We demonstrated that only 2.2% of CD4^+^ T cells expressed CD25 at the beginning of reperfusion. After 1 hour of reperfusion, the proportion of CD4^+^CD25^+^ T cells increased a little up to 2.8%. After 6 hours of reperfusion, the level of CD4^+^CD25^+^ T cells increased up to 8.2% and it dramatically came to 20.6% after 12 hours of reperfusion (Figure 1).

### Anti-CD25 mAb attenuates liver IRI

**Figure 2:**
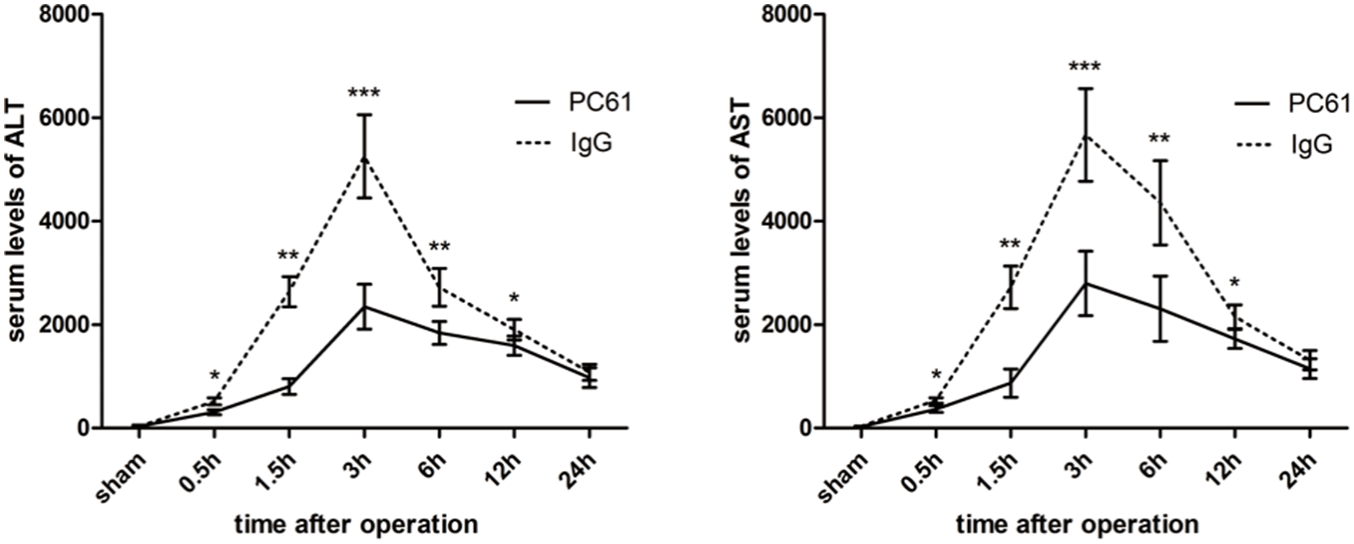
Anti-CD25 mAb pretreatment preserved liver function after hepatic IR. Serum ALT and AST levels in IgG and PC61 group (n = 6) after partial ischemia and 0.5, 1.5, 3, 6, 12, 24 hours reperfusion. Sham controls (n = 3) displayed blood sample at 3 hours after reperfusion. Results are mean values±SE. (*p<0.05, **p<0.01, ***p<0.001).

**Figure 3:**
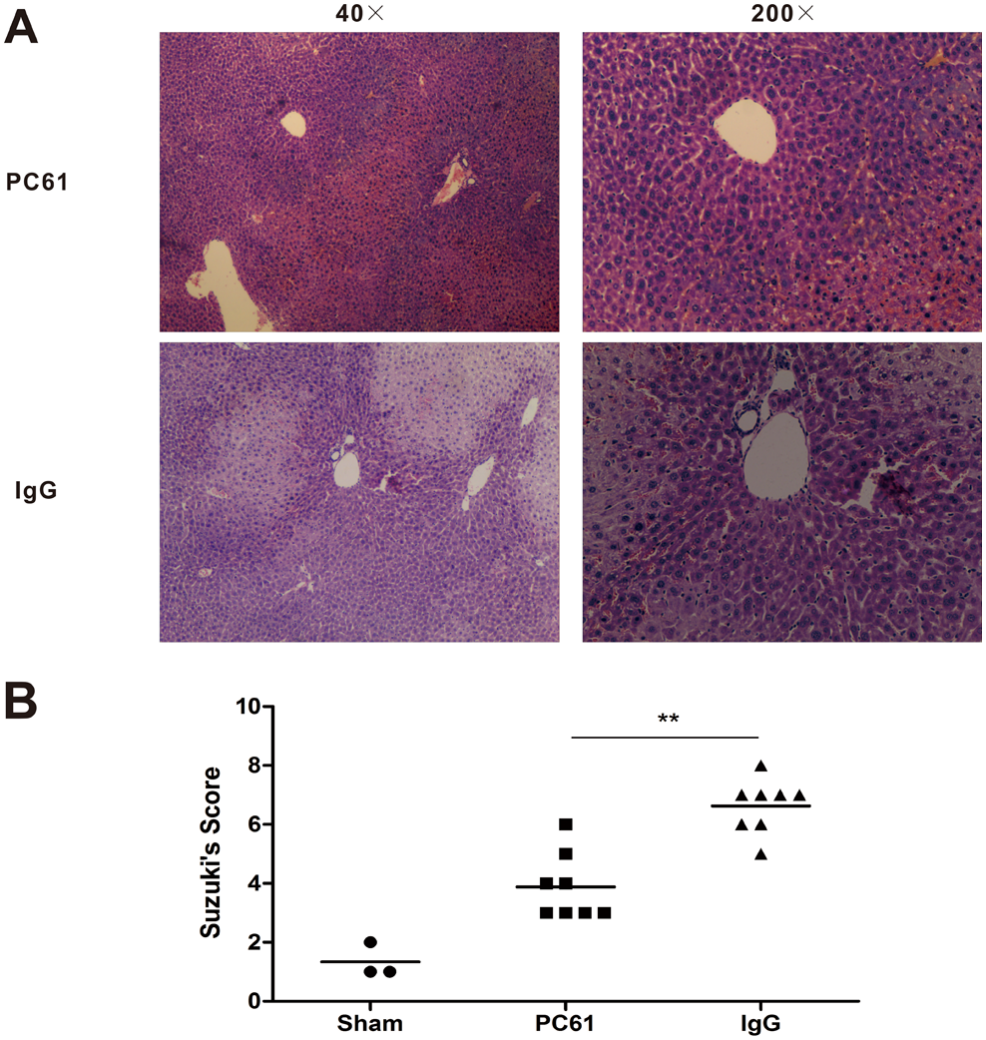
Anti-CD25 mAb pretreatment preserved liver function after hepatic IR. (A) Hematoxylin and eosin staining of liver right lobe harvested at 6 hours post reperfusion (40×, 200× original magnification). Sections are representative of 6 independent mice per group. (B) Scoring of the ischemic liver sections according to the Suzuki’s criteria. (*p<0.05, **p<0.01, ***p<0.001).

To assess the impact of anti-CD25 mAb on hepatic IRI, we first induced warm hepatic IR using a well-established two-lobe IR model, and different groups were subjected to 60 minutes of partial hepatic ischemia. At different time points post reperfusion, serum ALT and AST concentrations were collected and showed in Fig. 2. Those for sALT and sAST were significantly higher in the IgG group than in the PC61 group, which peaked at 3 hours postreperfusion (7282.3±1070.59 versus 2332.7±441.7 [P<0.001] for sALT, and 7707.6±1167.3 versus 2987.7±814.7 [P<0.001] for sAST). The serum transaminases levels remained statistically significant at 12 hours postreperfusion ([P = 0.024] for sALT, and [P = 0.016] for sAST) and had no statistical significance at 24 hours postreperfusion ([P = 0.314] for sALT, and [P = 0.174] for sAST) (Figure 2). Morphometric analysis of the percentage of hepatocellular necrosis confirmed difference in the level of hepatic injury at 6 h post reperfusion in PC61 group and IgG group (Fig. 3A). Large necrotic areas were evident in IgG group livers, whereas the hepatic architecture of PC61 group livers was better preserved with small and nonconfluent necrotic areas. Hepatocellular damage was graded according to Suzuki’s criteria (score, 6.63±0.47 compared to 3.97±0.44 in anti-CD25 group, P<0.01) (Fig. 3B).

### The suppressed proliferation of CD4^+^ T cells induced by PC61 is critically involved in liver IRI

**Figure 4:**
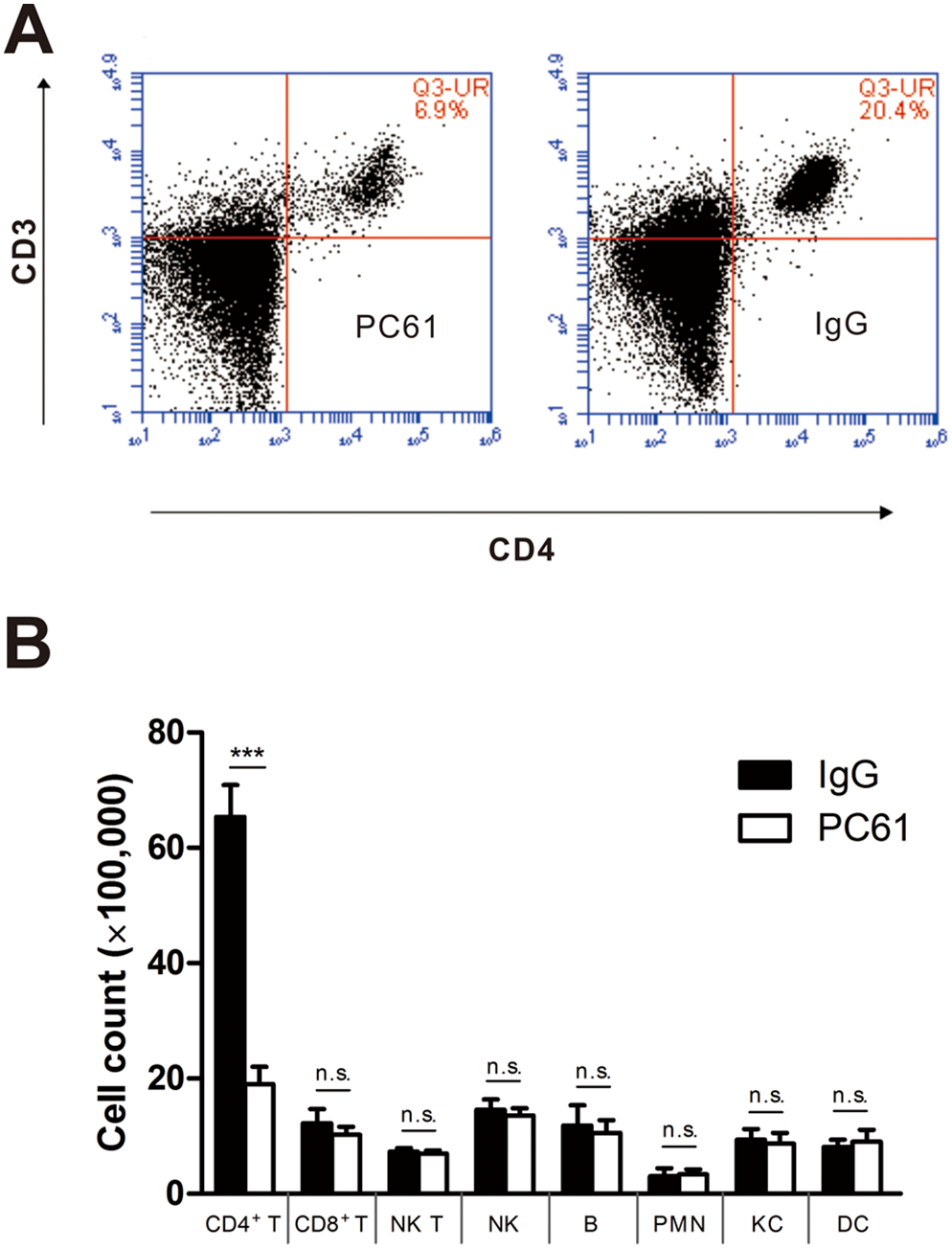
Anti-CD25 mAb administration reduced CD4^+^ T cells in mouse liver, not in blood or spleen. After one hour reperfusion, fleshly obtained hepatic nonparenchymal cells were isolated from PC61 or control IgG administrated C57Bl/6 mice. (A) Representative flow cytometry results from anti-CD3 and anti-CD4 stained hepatic nonparenchymal cells. (B) Total cells were gated on the 7-AAD^−^CD45^+^ before the FACS analysis, CD4^+^ (CD3^+^/CD4^+^), CD8^+^ (CD3^+^/CD8^+^) T cells, NK (CD3^–^/NK1.1^+^), NK T(CD3^+^/NK1.1^+^) cells, B cells (B220^+^/CD19^+^), neutrophils (GR-1^+^/CD11b^+^), Kupffer cells (F4/80^low^/CD11b^+^), or dendritic cells (F4/80^high^/CD11b^+^) were compared in each group. Data are mean values±SE; n = 5 per group. (***p<0.001).

The data presented thus far indicate that near-term anti-CD25 treatment is protective in the early phase of liver IR injury. To document the role of CD4^+^ T cells in liver inflammatory-mediated IR injury response, the effect of near-term PC61 administration versus IgG was determined by comparing the count of CD45^+^ cells 1 hours after reperfusion. PC61 treatment significantly decreased the percentage and number of CD4^+^ T cells (Fig. 4A). No significant effect of PC61 administration was observed on liver CD8^+^ T cells, B cells, neutrophils, Kupffer cells, dendritic cells, natural killer (NK) or NK T cells (Fig. 4B). In the blood and spleen, the number of lymphocytes and the proportion of CD4^+^, and CD8^+^ T cells did not change significantly during the first 12 h of reperfusion both in PC61 group and IgG group (data not shown).

**Figure 5:**
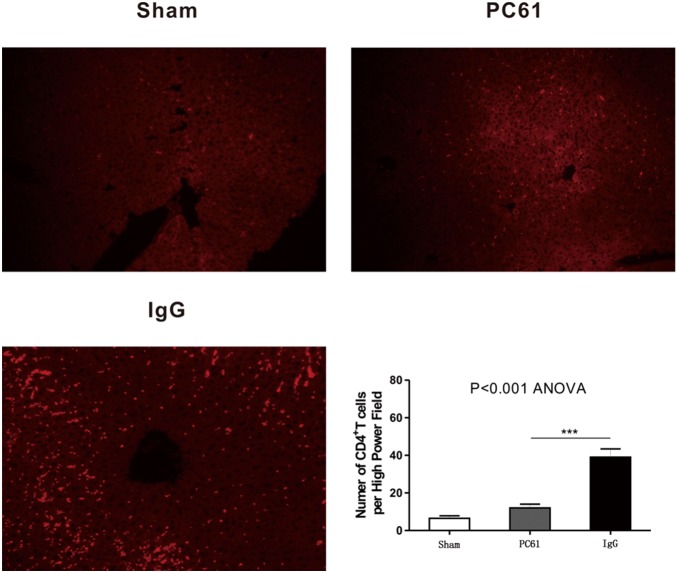
Anti-CD25 mAb pretreatment decreased CD4^+^ T cells proliferation and deposition. Immunofluorescence analyses of Sham, PC61, and IgG livers one hour following sham or partial hepatic ischemia-reperfusion surgeries. CD4 was stained red and the number of CD4^+^ T cells per High Power Field was figured up and quantitative analyzed.

Previous results suggested that PC61 inhibits CD4^+^ T cell proliferation, which plays a critical role in the process of IR-induced inflammatory. Liver immunofluorescences staining also got a similar result. The number of CD4^+^ T cells in both PC61 group and IgG group increased at 1 hours after reperfusion compared to sham group, and the accumulation of CD4^+^ T cells around the sinusoidal area was diminished by PC61 treatment (Fig. 5). This result likely reflected that the decreased expansion and deposition of CD4^+^ T cells resulted from the suppression function of anti-CD25 mAb.

### Anti-CD25 mAb mitigates intrahepatic inflammatory milieu after injury and blocks the positive feedback amplification

**Figure 6:**
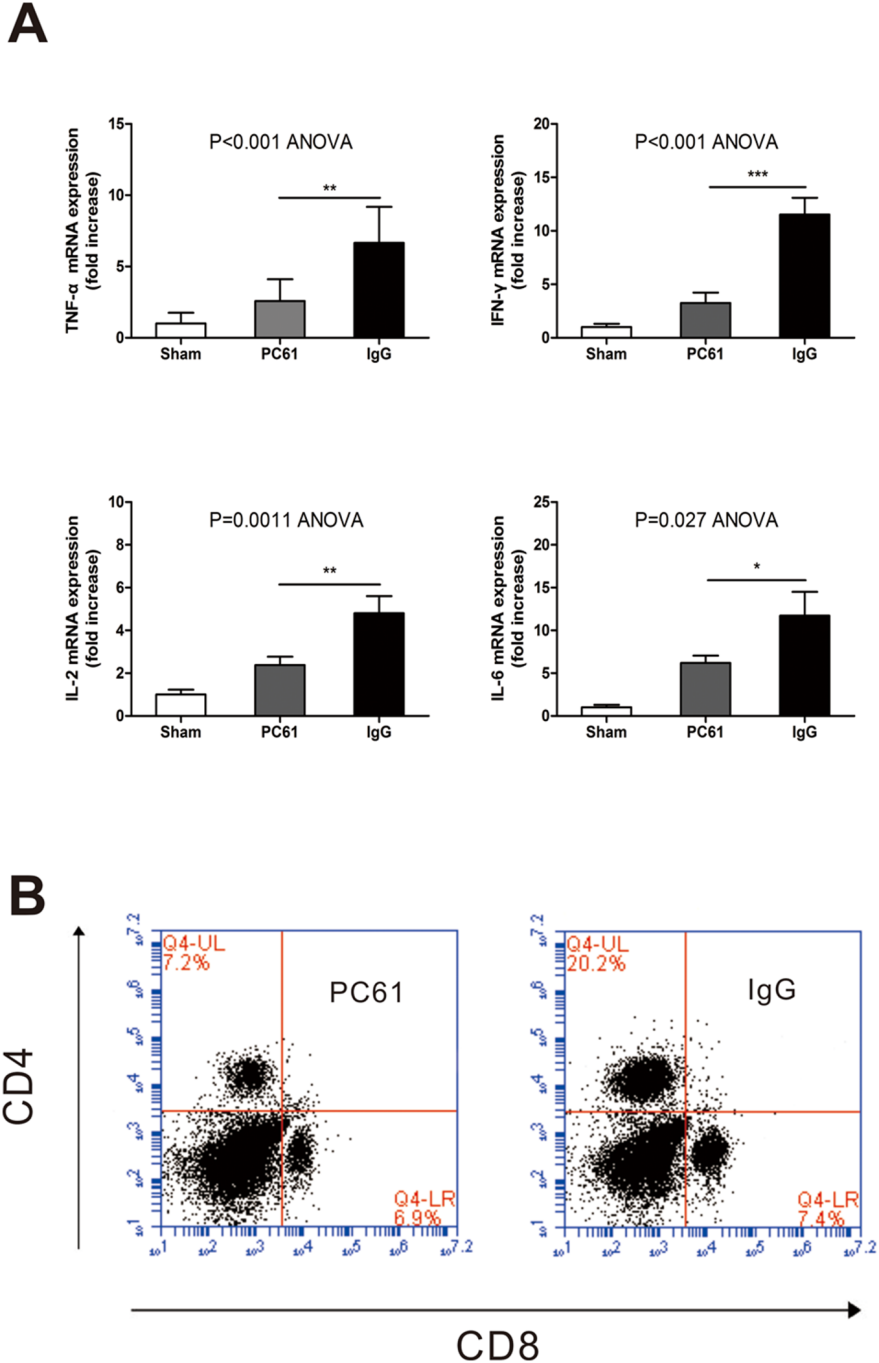
Anti-CD25 mAb pretreatment mitigated intrahepatic inflammatory milieu. (A) TNF-α, IFN-γ, IL-2, and IL-6 mRNA expression among sham, PC61, and I/R group after hepatic ischemia and 60 min of reperfusion. Samples were analyzed by RT-PCR. Data are expressed as means±SE; n = 6 per group. (B) Splenocytes were labeled with 5 mM 5-(and-6)-carboxyfluorescein diacetate, succinimidyl ester and co-cultured with samples’ protein in 5% CO_2_ at 37°C. After 72 hours proliferation was assessed by flow Cytometry. (*p<0.05, **p<0.01, ***P<0.001).

T cell activation involves antigen-dependent and –independent pathways [13]. Antigen-independent T cells activation definitely plays a pivotal role in liver IRI, and cytokines (including TNF-α, IFN-γ, IL-2, IL-6) have all been demonstrated to activate T cells directly [14], [15]. Meanwhile, activated T lymphocytes secrete kinds of proinflammatory cytokines that constitute a positive feedback loop in exacerbating liver IRI. Given the fact that cytokines play a critical role in IR-induced liver injury, we therefore compared their expressions between PC61 treated mice and IgG treated mice. Interestingly, mRNAs for TNF-α, IFN-γ, IL-2, IL-6 were significantly higher in I/R mice as compared with that of PC61 treated mice (Fig. 6A), revealing that anti-CD25 mAb inhibits IR-induced aseptic inflammation in the livers. To demonstrate whether the reduced cytokines level relevant to PC61 pretreatment is associated with T lymphocytes’ proliferation, we harvested splenocytes from naïve C57Bl/6 mice and incubated these splenocytes with samples’ protein from two groups in 5% CO_2_ at 37°C. As expected, the percentage of CD4^+^ T cells of IgG group was significantly higher after 72 hours cultivation compared with that of PC61 group (Fig. 6B).

### Near-term anti-CD25 mAb administration has no effect on Treg mobilization or expansion

**Figure 7:**
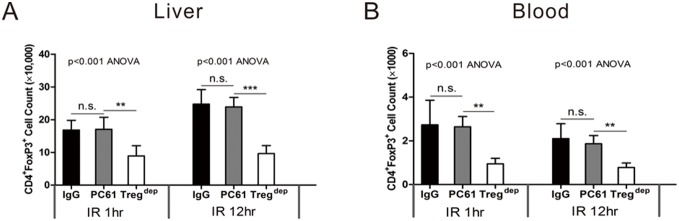
The number of Tregs did not change significantly when anti-CD25 mAb were administered shortly before IR insult. After 1 hour or 12 hours of reperfusion, total hepatic nonparenchymal cells and spleen cells were isolated and gated on 7-AAD^−^CD45^+^ before the analysis. Tregs were identified as CD4^+^/FoxP3^+^. Data are mean values±SE; n = 6 per group. (n.s. P>0.05 **P<0.01, ***P<0.001).

Importantly, despite the significant protection from injury offered by anti-CD25 mAb, it is also known as a depletion antibody of Tregs. To investigate the role of Tregs, we adopted a classic Treg depletion model (PC61 300 µg intravenously 5 d before IR). This dosing strategy was chosen on the basis of the kinetic analysis of single-dose PC61 on FoxP3^+^ Tregs in C57Bl/6 mice [16]. Absolute numbers of CD4^+^FoxP3^+^ Tregs were detected 1 hour and 12 hours after reperfusion. As expected, the total number of CD4^+^FoxP3^+^ Tregs was significantly decreased in the Treg depletion mice both in liver and blood. Interestingly, although Tregs experienced an increase in liver and a decrease in blood in a time dependent manner, the near-term administration of PC61 did not modify the number of Tregs significantly. (Fig. 7).

## Discussion

Liver ischemia-reperfusion injury occurs in the clinical settings of hepatic resection surgery, hemorrhagic trauma, and liver transplantation. In the last 20 years, the rates of acute and chronic rejection have fallen dramatically, for example, the incidence of acute rejection during the first six months post-transplant has declined from over 40% in 1995 to around 15% in 2000 [17]. Part of this improvement results from increased use of selective induction agents, particularly the interleukin-2 receptor antagonists (anti-CD25 mAb). Addition of anti-CD25 mAb to a variety of calcineurin inhibitor-based immunosuppressive regimens reduced acute rejection by 30–50% as reported in two meta-analyses of the randomized controlled trials. These meta-analyses also demonstrated trends toward improved graft survival with anti-CD25 mAb compared with no induction [18], [19]. Although the protective effect for induction therapy of anti-CD25 mAb has been widely documented in liver transplantation field, the exact effect of anti-CD25 mAb on liver IR injury, however, remains poorly elucidated [20]–[23].

The present study for the first time demonstrated that anti-CD25 mAb administration shortly antecedent to liver IR induction provides protection in the initiation phase of injury. After 70% liver ischemia, anti-CD25 mAb pretreated mice displayed significantly preserved liver function as characterized by less histological damage and reduced serum enzymes level. We further demonstrated that the protective effect was associated with ameliorated intrahepatic inflammatory milieu and reduced CD4^+^ T lymphocytes as manifested by the decrease of proinflammatory cytokine production (less expression of TNF-α, IFN-γ, IL-2, and IL-6) and the lower CD4/CD8 proportion. By employing an *in* *vitro* lymphocytes proliferation assay model, we confirmed that the protective effect of near-term antecedent anti-CD25 mAb treatment on IR-induced liver injury depend on the inhibition on the proliferation of CD4^+^ T lymphocytes. Taken together, our data demonstrated that near-term antecedent anti-CD25 mAb treatment provides protection for livers against subsequent IR-induced injury by inhibiting the proliferation of CD4^+^ T lymphocytes and mitigates intrahepatic inflammatory milieu through decreasing the proinflammatory cytokine production.

Our data presented in the current report are in agreement with previous studies in the lung, kidney and brain after IR induction [4], [24]–[26]. Using a lung transplantation model, Marc de Perrot et al, demonstrated that early reperfusion injury is dependent of donor but not recipient T cells, and recipient CD4^+^ T cells infiltrated lung grafts within 1 hour of reperfusion. When compared to T cell-deficient nude rats (rnu/rnu), WT rats had decreased oxygenation and increased peak airway pressures indicating more severe injury in the mice with functional T cells [25]. In another renal IR injury model, both nu/nu mice and CD4^−/−^ mice had significantly less histopathologic and serologic injury compared to WT mice. CD8^−/−^ mice had similar functional and structural injury patterns as seen in WT mice. Reconstitution of nu/nu mice by adoptive transfer with T cells from WT mice restored the injury phenotype as did adoptive transfer of isolated CD4^+^ T cells into the CD4^−/−^ mice [4]. Interestingly, we observed a similar protective pattern against hepatic IR injury in anti-CD25 mAb antecedent treated mice. We found that anti-CD25 mAb pretreatment reduced hepatocyte injury in the early phase during 24 hour after IR insult, and more powerful in the initiation phase at 3 hour after reperfusion. Meanwhile, the number of CD4^+^ T cells in liver after IR insult was significantly lower in the PC61 group than that in the IgG group, but no significantly change was found in the blood and spleen during the first 12 hours of reperfusion. These results suggested that this brisk response, which preceded the influx of innate immune cells to the injured tissue, is mainly associated with resident T lymphocytes. These organ-resident T lymphocytes can maintain the expression of specific cytokine receptor on their cell surface and proliferate *in* *vivo* in the absence of Ag stimulation [27].

It has been previously demonstrated that use of MHC-II-blocking antibodies has no effect on serum alanine transaminase following hepatic IR, which suggested that T cells play a role not involving the αβ TCR and that lymphocyte actions occur through a non-antigenic mechanism [28]. Furthermore, non-naïve as well as unconventional T cells can be functionally activated by cytokines in a manner independent of TCR engagement [29], [30]. These studies proposed that cytokines produced during I/R might directly activated CD4^+^ T cells. In line with this result, we found that the proportion of CD4^+^ T cells in our model was associated with expression of cytokines. Our *in* *vitro* studies demonstrated that the proportion of CD4^+^ T cells was markedly elevated in inflammatory milieu.

Treg is known as a critical role in maintaining immune homeostasis. In the model of IR injury, Treg functions to restrain excessive Teff cell responses [12], [31]. In line with the studies of depletion kinetics of PC61, the near-term administration of PC61 did not modify the number of Tregs significantly, and the depletion of CD4^+^FoxP3^+^ Tregs in PC61-treated mice was already apparent 4 days after injection [32]. At the same time, since IL-6 acts as a potent proinflammatory cytokine and has the ability to inhibit Treg differentiation, higher level of IL-6 may also contributed to the limited expansion of Tregs in IgG-treated mice compared with PC61-treated mice in liver [33].

Although the role of CD4^+^ T cells in IR injury has been noted as early as 1997 (by Zwacka [34] *et al*), there are still some obstacles to using what we know about the depletion of CD4^+^ T cells in clinical applications. On one hand, depletion of CD4^+^ T cells leads to progressive impairments in cellular immunity and increases the susceptibility to opportunistic infections, as manifested by the effects of HIV infection whose hallmark is the progressive depletion of CD4^+^ T cells. On the other hand, regulatory T cells (Tregs), one of the CD4^+^ T-cell subsets, are commonly known to have immunosuppressive properties, and are known to have the ability to circulate to areas of inflammation and thereby mitigate immune reactions [35], [36]. Therefore, the depletion of CD4^+^ T cells will abrogate the protective effect of the Tregs. The data we reported in our manuscript resulted from a hepatic IR mode, which provides the first line of experimental evidence that demonstrates the effect of the near-term antecedent anti-CD25 mAb treatment for the protection of the hepatic against IR-induced injury in the early phase (24 h) after IR insult. Moreover, our work suggests that the anti-CD25 mAb simply inhibited the proliferation of the hepatic resident CD4^+^ T cells rather than influenced the CD4^+^ T cells in the blood or the spleen. Also, it did not modify the number of Tregs found in the liver or the blood. Altogether, our results demonstrate a potential function of the anti-CD25 mAb, which was neglected in the past, and may be helpful for redesigning the usage and dosage of anti-CD25 mAb treatments in various clinical conditions, particularly in liver and kidney transplantations.
